# Creative Lockdown? A Daily Diary Study of Creative Activity During Pandemics

**DOI:** 10.3389/fpsyg.2021.600076

**Published:** 2021-02-09

**Authors:** Maciej Karwowski, Aleksandra Zielińska, Dorota M. Jankowska, Elzbieta Strutyńska, Iwona Omelańczuk, Izabela Lebuda

**Affiliations:** ^1^Institute of Psychology, University of Wrocław, Wrocław, Poland; ^2^Institute of Education, The Maria Grzegorzewska University, Warsaw, Poland

**Keywords:** daily diary, creativity, multilevel modeling, COVID-19, emotions

## Abstract

The coronavirus disease 2019 (COVID-19) pandemic is influencing our lives in an enormous and unprecedented way. Here, we explore COVID-19-lockdown's consequences for creative activity. To this end, we relied on two extensive diary studies. The first, held on March 2019 (pre-pandemic), involved 78 students who reported their emotions and creativity over 2 weeks (927 observations). The second, conducted on March 2020 (during the pandemic and lockdown), involved 235 students who reported on their emotions, creativity, and the intensity of thinking and talking about COVID-19 over a month (5,904 observations). We found that compared with 2019, during the lockdown, students engaged slightly yet statistically significantly more in creative activities. An analysis of diaries collected during the pandemic also showed that the days when students spent more time discussing or searching for information about COVID-19 were characterized by a higher creative activity yet also mixed emotions. We discuss potential explanations of these unexpected results along with future study directions.

## Introduction

The current pandemic created an entirely new reality for almost anyone across the world. While its influence on creativity has only recently begun to be analyzed (Beghetto, 2020; Kapoor and Kaufman, 2020), emphasizing both opportunities (Kapoor and Kaufman, 2020; Orkibi, 2020) and challenges (Karwowski et al., 2020) of the current situation, the devastating effects of the lockdown on creative professions have already been recognized. As the Creative Industries Federation called it (June 17, 2020), what coronavirus disease 2019 (COVID-19) caused is a “cultural catastrophe” (Creative Industries Federation, 2020). Indeed, musicians, actors, or visual artists in most countries struggle seriously, as their possibilities to work are almost entirely undermined (see Comunian and England, 2020). However, what is less known is the impact of pandemics on less professionalized creativity, primarily the so-called mini-c or little-c—to use the Four-C model's terms (Kaufman and Beghetto, 2009). The Four-C model distinguishes different forms and aspects of creativity, starting from the mini-c creativity (primarily cognitive processes engaged in learning) and little-c creativity (problem-solving and everyday creativity) to pro-c (professional creativity, usually conducted within a certain domain) and Big-C creativity—typical for very few geniuses. This investigation focuses on creative activity, so the little-c level of creativity.

Do we think or act creatively to the same extent as prior to pandemics? Does the combination of uncertainty and danger caused by pandemics make us more or less creative? In other words, can the situation caused by COVID-19 motivate and drive individuals to produce something creative? Apparently, it worked for Isaac Newton, who created major new insights for his theories of gravity and optics during enforced isolation caused by the outbreak of the Great Plague of London. Yet anecdotes aside, what are the effects of the pandemic on cognitive processes relevant to creativity and everyday creative behavior? While the former aspects of creativity in relation to a pandemic were already researched, this study focuses exclusively on creative activity.

### Creativity and the Pandemic

It has already been demonstrated that when people are primed to think about coronavirus, they become less tolerant (Sorokowski et al., 2020) and more conservative (Karwowski et al., 2020). Also, research on the need for closure has shown that under conditions of diffuse uncertainty, people choose simplistic solutions, have a tendency to think in extremes, and exhibit dichotomous thinking (e.g., Webster and Kruglanski, 1994). It quite naturally leads to expecting detrimental effects for creativity, given that creative people are usually tolerant (Groyecka, 2018) and liberal rather than conservative (Dollinger, 2007) and use a variety of creative thinking strategies (Jankowska et al., 2018). What is more, there is evidence that thinking about COVID increases the level of stress (Kowal et al., 2020), a factor negatively related to creative thinking (Duan et al., 2020). A recent study that tested whether activating thinking about coronavirus influences people's insight and analytical problem-solving (Karwowski et al., 2020) found a negative effect, yet only among men. Even more interestingly, this study also demonstrated that the participants who were primed with information about coronavirus tended to be more careless and less engaged in the tasks presented. This result suggests that when thinking about coronavirus is activated, people might become focused and task-oriented yet less likely to engage in creative thinking, driven primarily by enjoyment (Benedek et al., 2020) and intrinsic motives (Anderson and Karwowski, 2020).

One of the possible mechanisms that might explain the expected negative influence of COVID-19, and associated lockdown, on creativity is the effect of emotions. The pandemic situation is emotionally challenging; thinking about COVID-19, not to mention the lockdown and threat caused by the risk of illness, increases sadness, stress, anger–hostility moods and reduces positive emotions (e.g., Pérez-Fuentes et al., 2020; Shanahan et al., 2020). The role of emotions for creativity is well-established, with decades of research showing quite consistently that positive, activated, and promotion focused emotion, such as feeling happy, enthusiastic, and elated, improve creative performance (Baas et al., 2008; Davis, 2009).

Also, a growing number of dynamic micro-longitudinal studies show clear links between positive emotions and creativity. An experience-sampling study found that better advancement in the creative process in art was supported by the enjoyment of work and hampered by work-related anxiety (Benedek et al., 2017). A diary study in work settings showed a positive linear relationship between positive affect and creativity (Amabile et al., 2005). In the experience-sampling study among young adults, everyday creativity was linked to feeling happy and active (Silvia et al., 2014; see also Conner and Silvia, 2015). A study that simultaneously applied the experience-sampling method and day reconstruction method found that active, positive emotions (happiness, concentration, feeling engaged, and interested) positively correlated with creativity in everyday life among corporate employees (Han et al., 2019). The benefits of a positive mood on creativity were corroborated in research with middle-aged participants that utilized both experience sampling and diary methods (Karwowski et al., 2017). Yet this study also found that some negative emotions, such as feeling restricted and unfocused, predicted everyday creativity as well (Karwowski et al., 2017). At the same time, however, causal links are still unclear. For example, in their large (*N* = 658) daily diary study, Conner et al. (2018) provided a convincing argument that it is creative activity that leads to flourishing (operationalized as positive affect) than vice versa. Hence, if creativity builds positive emotions rather than positive emotions build creativity, then reasoning about the relationship between COVID-19 and creative activity becomes even more complex. If positive emotions do not causally influence creativity, they cannot be responsible of (and thus—mediate) the links between COVID-19 and creative activity.

The role of negative mood for creativity is far less clear, and contextual conditions likely moderate it (e.g., Van Kleef et al., 2010). While positive mood seems to support early idea generation, neutral and negative emotions enhance performance in later idea production (Kaufmann and Vosburg, 2002). Importantly, anger was found to be beneficial in early stages of the creative process (Baas et al., 2011). The creative task itself is an essential piece of the puzzle. When people frame a task as fun, they produce more ideas in a positive mood, but when they frame a task as serious, they are more efficient in a negative mood (Friedman et al., 2007). Moreover, negative moods can make people more critical and discerning, whereby they produce ideas that are not only creative but also useful (George and Zhou, 2002).

Therefore, creativity might benefit from a mix of positive and negative emotions, a situation we currently observe. Information about COVID-19 might be perceived as a threat to valuable resources like work, health, or even life (Hobfoll, 2001) and, in consequence, evoke stress, fear, and anxiety. But in these stressful conditions, all positive information, like new scientific discoveries announced, gains particular importance (Hobfoll, 2001; Hobfoll et al., 2018) and could raise hope, engagement, and interest. Additionally, using effective strategies for coping with stress can help individuals cope with the mental health challenges of quarantines and build positive emotions during COVID-19 social distancing (Fischer et al., 2020). This coexistence of different emotional states plays a role in creativity, as does the affective shift (Bledow et al., 2013). Experiencing mixed emotions works similarly to getting conflicting information; it informs about unusual circumstances and motivates to generate uncommon ideas (Fong, 2006). When people feel happy along with feeling tired, they more easily accept unusual ideas and solutions (Middlewood et al., 2016). When they feel happy and sad simultaneously, they are more open to new, alternative perspectives (Rees et al., 2013) and score higher on creative thinking (Fong, 2006). However, it is worth to keep in mind that people have difficulty recalling the mixed emotions experience after some time and in the long run remember them as less diverse (Aaker et al., 2008). To prevent this memory decline of mixed feelings, it could be useful to employ the in-the-moment emotions assessment (see Moeller et al., 2018).

### Creative Thinking vs. Creative Activity

Our overview quite naturally leads to the expectation that thinking about COVID-19 will reduce positive emotions and increase negative ones, particularly anxiety and fear. Consequently, in the short term, COVID-19—as any other threat—is expected to diminish creative thinking and problem-solving. What is less clear, however, is what the medium-or-long-term consequences of COVID-19 are for creative activity and behavior. Lockdown caused by the pandemic forced people to stay home, so the open question is what the influence of this lockdown is for their creative functioning. However, before we answer this, we should ask what faces creative activity of emerging adults might have.

Different expressions of a creative potential during adolescence and early adulthood usually fall into the broad category of everyday or little-c creativity (Kaufman and Beghetto, 2009). They can be seen as a springboard for professional and eminent creative accomplishments. Importantly, however, creative actions in which the nonexpert, the ordinary person, participates on a daily basis are more than merely a stage in creative development. Everyday creative acts such as telling a joke and making people laugh or taking photographs bring originality and meaningfulness into one's life and improve personal well-being (Ivcevic, 2007; Richards, 2010). So if the pandemic makes people's creative activity lower, this is by no means a trivial thing. But if during the pandemic people engage in creative activity more often—be it because of boredom, more time for hobbies, or a conscious decision to focus on meaningful activity—the pandemic could have its paradoxically positive face as well.

Young adults express a variety of creative behaviors in their everyday life (Silvia et al., 2014; Conner and Silvia, 2015; Zielińska, 2020). The originality and usefulness of these acts can be recognized by the creator or at most a small circle of friends and relatives. This private nature of everyday creativity differentiates it from the more formal domains of work, in which the experts' recognition and its broadness are the core characteristics of a creative pursuit (Carson et al., 2005). Furthermore, although successful performance as a professional creator requires specialized training and know-how, creativity in daily life is not conditioned by specific skills and knowledge. It is universal, is expressed by many people, and has an adaptive function (Ivcevic and Mayer, 2009; Richards, 2010).

In late adolescence and emerging adulthood [see Karwowski and Wiśniewska, (in press)], people become more aware of their creative abilities and begin to perceive creativity as an essential aspect of the self (Karwowski, 2016). They develop creative aspirations, transform them into creative identities, and foster the competencies useful in a particular area of creative activity (Zielińska, 2020). Starting from exploration, i.e., engaging in such forms of creative activity that interest them, young people gradually focus their commitment in a specific domain, which in turn leads to the differentiation of their creative potential, that is, the development of domain-specific skills and abilities (Barbot and Tinio, 2015).

## The Present Study

The present study benefits from two daily diary studies conducted on two similar samples on March 2019 (before the pandemic) and on March 2020 (during the pandemic, including the most restrictive lockdown). Two main questions drive our endeavors here. On the most general level, we are interested in the possible effects that the lockdown associated with COVID-19 might have on creative activity. Although it might be posited that being at home, sometimes probably bored, creates a unique opportunity for creative endeavors (e.g., Gasper and Middlewood, 2014), previous research suggested the opposite pattern—a negative effect of pandemics on creative activity—likely due to growing anxiety (and negative emotions in general) and reduced positive emotions. We explore this effect in a two-fold manner: first by comparing creative activity in 2019–2020 and by examining whether the intensity of information about COVID-19 (in 2020 only) is associated with creative activity at the level of people (level 2) and days (level 1).

## Method

### Participants

A total of 313 first-year University students participated in this study in 2019 and 2020. There were 78 students (65 female) in 2019 and 235 in 2020 (213 female). All participants were social sciences students (psychology or education). Students volunteered to participate and received credit toward a research option in their class. In 2019, the participants filled daily diaries over 13 days (February 27 to March 12); in 2020, they filled diaries over 31 days (from March 19 to April 18th). Poland's universities were closed from March 12 to the end of the semester (June), with a strict lockdown being announced by the government between March 25 and April 19. To increase intra-individual variability (day level), we excluded students who participated in a study (*n* = 60 total) but filled < five diaries. The total number of level 1 (*days* × *person*) data was 927 for 2019 and 5,904 for 2020.

### Measures

We used the same core measures in 2019 and 2020, with additional items explicitly added in 2020 to capture information-seeking about COVID-19.

#### Creative Activity

Every day, the participants rated the intensity of their engagement in 15 different activities, using a 7-point Likert scale (1 = *not at all*, 7 = *very intensively*). The activities were adapted from previous studies (Karwowski and Brzeski, 2017; Karwowski et al., 2017) and are presented in Supplementary Table 1. They resulted in a reliable scale when averaged (α on level 2 = 0.81, on level 1 = 0.67).

#### Emotions

Each day, participants rated how strongly they felt each of the 21 presented emotions during the day. This scale was taken from Karwowski et al. (2017) and captured emotions that resemble those typically used to measure positive and negative affect within the circumplex model of affect (Barrett and Russell, 1999). Participants used a 7-point Likert scale with 1 = *not at all*, 7 = *very often*. Among these emotions, three were particularly related to anxiety, so we also analyzed them separately (i.e., how often they felt afraid, nervous, and concerned on that day). The complete list of emotions, together with descriptive statistics, is presented in Supplementary Table 1.

#### Information About Coronavirus Disease 2019

In 2020, there were two additional daily questions about participants' exposition to information about COVID-19: how intensively they searched for information about coronavirus and how much time they spent talking about it (the participants used a 7-point Likert scale, ranging from *1* = *I did not do it at all* to *7* = *I did it very intensively*). We averaged scores across the two questions to obtain a single measure of the exposition to information about the coronavirus (α on level 2 = 0.90, on level 1 = 0.83).

### Procedure

After responding to the invitation to participate in a study, participants completed the informed consent form. They completed an online daily diary accessible between 6:00 p.m. and 11:00 p.m. The procedure was the same for 2019 and 2020, except that there were some additional variables measured in 2020 (specific to the situation), and the 2020 module of the study lasted for a month.

### Data Analysis

As our data had a hierarchical structure, with days nested within persons, we relied on multilevel modeling in Mplus 8.1 (Muthén and Muthén, 1998–2017). Our main analyses were conducted in two steps. First, to compare 2019 with 2020, we examined level 2 effects of the year on emotions and creative activity. Our predictor (year: 2019 vs. 2020; lockdown or lack of it) was a dichotomous, level 2 variable. Our second step, conducted only on the 2020 data, examined whether there are links between the intensity of information about COVID-19, positive and negative emotions, and creative activity. To test for possible causal effects, this analysis was complemented with lagged analyses.

### Ethic Statement

All subjects gave informed consent in accordance with the Declaration of Helsinki. The protocol was approved by the University of Wroclaw's Institute of Psychology Institutional Review Board.

## Results

### Data Reduction and Descriptive Statistics

As presented in Supplementary Table 1, there was a clear two-factor solution for emotions suggested by a parallel analysis, with positive and negative emotions factors, which were saved for further analyses. Table 1 presents correlations between variables, both between- and within-person, for the total sample (2019 and 2020 aggregated). Intriguingly, the amount of information about COVID-19 was positively related not only to negative emotions and anxiety (as we predicted) but also to positive emotions (*r* = 0.12 within-level and *r* = 0.13 between-level).

**Table 1:** Level 1 and level 2 correlations between variables of the study.

		**1**	**2**	**3**	**4**	**5**
1	Info COVID-19	1	**0.12**	**0.20**	**0.23**	**0.20**
2	Positive emotions	**0.13**	1	**−0.18**	**−0.08**	**0.33**
3	Negative emotions	**0.27**	−0.07	1	**0.86**	**−0.03**
4	Anxiety	**0.32**	−0.03	**0.91**	1	0.01
5	Creative activity	**0.25**	**0.41**	0.01	0.06	1

*Correlation on the day level (N = 6,823, except for correlations for the first variable, Info COVID-19, N = 5,917) are above the diagonal. Correlations on the person level (N = 313, except for correlations for the first variable, Info COVID-19, N = 236) are below the diagonal. Statistically significant (p < 0.05) correlations are in bold*.

*COVID-19, coronavirus disease 2019*.

Before starting our multilevel analyses, we estimated variability of each of the variables of interest, associated with a person (level 2) and a day (level 1). Intraclass correlation coefficients (ICCs) are presented in Table 2. In all cases there was substantial day-to-day variability—a finding that supports our decision to use multilevel models.

**Table 2:** Intraclass correlation coefficients estimated in empty models separately for 2019 and 2020 study.

	**2019**	**2020**
Info COVID-19	–	59%
Positive emotions	35%	42%
Negative emotions	32%	47%
Anxiety	26%	43%
Creative activity	52%	58%

*COVID-19, coronavirus disease 2019*.

### Main Analyses

Our first model explored cross-sectional differences in emotions and creativity among students who participated in our study in 2019 and 2020. We emphasize that although the design was the same (apart from a more prolonged study in 2020), these comparisons are cross-sectional, based on different participants, so any causal conclusions would be premature. Still, it seemed interesting and relevant to start with such a comparison given similarity of samples and measures.

As illustrated in Table 3, on average in 2020 (lockdown), students tended to report slightly less positive emotions than did participants in 2019 (no lockdown) (*p* = 0.02), yet there was no predicted higher intensity of negative emotions or anxiety in 2020. Intriguingly though, we found a statistically significant positive effect of the year (2019 vs. 2020) on creative activity.

**Table 3:** Effect of year on emotions and creativity (differences between 2019 and 2020).

	**Effect of year**		
	**β**	**95% CI**	***p***
Positive emotions	−0.11	– 0.26, −0.03	0.02
Negative emotions	−0.04	−0.16, 0.08	0.26
Anxiety	0.05	−0.07, 0.17	0.26
Creative activity	0.10	0.01, 0.21	0.03

*All values are standardized*.

The effect of the year (2019 vs. 2020 or no lockdown vs. lockdown) shows that students who participated in our study were more creatively engaged during the lockdown than their peers a year before. Given that this comparison is cross-sectional, our second step concerned the 2020 dataset only, with a particular focus on the information about COVID-19 as an independent variable.

For 2020 data, we estimated effects both within-person and between-person, to examine whether higher intensity of COVID-19-related news was associated with elevated negative and reduced positive emotions and how emotions and intensity of COVID-19-related news were linked to creative activity. On both person level and day level, we observed statistically significant and positive relationships between the intensity of COVID-19 news and negative emotions—an effect we had predicted. But we also found a positive relationship between the intensity of COVID-19 news and positive emotions—a finding contrary to our predictions. This effect was obtained on level 2, which indicates that people who were more engaged in searching and discussing COVID-19 tended to report both more negative (which is understandable) and more positive (which is more puzzling) emotions. Importantly, though, the same pattern occurred on the day level; the days when people discussed COVID-19 more intensively tended to be associated with more positive and more negative emotions. In our *Discussion*, we speculate why this could be the case. Here, we emphasize that this positive link between COVID-19 news and positive emotions was unexpected.

However, our main research question concerned the effect of COVID-19-related thinking on creativity. The surprising pattern obtained in year-to-year comparisons was even more pronounced when we focused on 2020. As Table 4 illustrates, on the person level, we observed that a tendency to discuss coronavirus issues more intensively or searching for COVID-19 related information was positively related to creative activity. On the day level, we found that days with a higher search for information about COVID-19 were associated with a higher creative activity as well. The relationships with positive emotions were consistent on the between- and within-level: the higher the positive emotions people declared, the higher their creative activity was, and the more positive the days they described, the higher their creative activity on these days was. There were no effects of generalized negative emotions nor anxiety on either person or day level.

**Table 4:** Creative activity as predicted by COVID-19 information search, positive and negative emotions on a person or day level.

	**Creative activity**		
	**β**	**95% CI**	***p***
**Person level**			
Info COVID-19	0.21	0.10, 0.33	<0.001
Positive emotions	0.43	0.33, 0.53	<0.001
Negative emotions	−0.04	−0.13, 0.08	0.29
Anxiety	−0.03	−0.12, 0.10	0.37
**Day level**			
Info COVID-19	0.11	0.09, 0.13	<0.001
Positive emotions	0.22	0.20, 0.25	<0.001
Negative emotions	0.00	−0.02, 0.03	0.47
Anxiety	0.00	−0.02, 0.03	0.46

*All values are standardized*.

### Lagged Analyses

Finally, to provide some insights into causal relationships, we conducted a series of lagged analyses (Nezlek, 2008, 2011). The first set of analyses tested lagged links from searching for information about the coronavirus to positive and negative emotions; the second tested the links from positive and negative emotions to searching for the information about the coronavirus. A separate set of analyses examined the links from emotions to creative activity and from creative activity to emotions. A lag was defined in terms of 1 day.

Our models provided arguments for reciprocal links between emotions and searching for the information about the coronavirus. Searching for information about the coronavirus was positively related to next-day positive (β = 0.07, 95% CI: 0.05, 0.09) and negative (β = 0.04, 95% CI: 0.01, 0.07) emotions. On the other hand, both positive (β = 0.05, 95% CI: 0.02, 0.08) and negative (β = 0.04, 95% CI: 0.01, 0.07) emotions predicted searching for information about the coronavirus next day. While the links were significant, we emphasize on their low effect size.

The second set of models tested the cross-lagged links between emotions and creative activity. Creative activity predicted positive (β = 0.06, 95% CI: 0.04, 0.09) but not negative emotions (β = 0.02, 95% CI: −0.004, 0.05) the next day. Neither positive (β = 0.01, 95% CI: −0.02, 0.04) nor negative emotions (β = 0.03, 95% CI: −0.01, 0.06) predicted creative activity next day. Lack of lagged links from emotions to creative activity replicates previous, equally well-powered diary findings (Conner et al., 2018). We emphasize here that the lack of causal links from positive emotions to creative activity does not preclude that positive emotions positively influence creative thinking.

## Discussion

COVID-19 devastates not only our health but also economies, including creative professions (Comunian and England, 2020). While its effects on everyday creativity are less established (but see Mercier et al., 2021), there is a growing number of studies that lead us to infer that even thinking about the threat associated with the recent pandemic is harmful to creative thinking and problem-solving (Karwowski et al., 2020). But does it apply to creative activity as well?

This study, utilizing data from unique pre-and-during-pandemics daily diary studies, searched for potential effects of COVID-caused lockdown on students' creative activity. We predicted that students locked in their houses will feel not only more negative emotions, anxiety in particular, but also that the confinement will harm their creative activity. What we observed, however, is not only contrary to our predictions but also apparently more complex.

In short, what both our micro-longitudinal studies—smaller and shorter in 2019 and more elaborated during the pandemic—demonstrated is exactly opposite to what we had expected. A cross-sectional comparison of 2019 and 2020 participants has shown that those who were locked in their homes had more, not less, opportunities for creative behaviors. While they declared lesser positive emotions in 2020 as compared with 2019, we did not observe differences in negative emotions and anxiety in particular.

When we dug deeper into the 2020 results, it became apparent that searching around for information about COVID-19 was positively linked with creative activity. Intriguingly, we also observed an unexpected effect of COVID-19 information on emotions. Not only higher saliency of COVID-19 was associated with negative emotions, but also—contrary to what we expected—it was related to experiencing more positive emotions, albeit with a weak effect size. Why did COVID-19 saliency link to a higher creative activity and positive emotions? We offer some interpretations below.

It is possible that our results serve as a unique demonstration of the conservation of resources theory (Hobfoll, 2001; Hobfoll et al., 2018). According to the “gain paradox principle,” when the chance for resource loss is high, any resource gains become more valuable. It is likely that in stressful circumstances of potential resource loss, such as decrease in well-being, for instance, young people invest more willingly in gaining other resources, such as developing their creative potential. It is also possible that broadening knowledge about the pandemic outbreak enhances the sense of control, which mediates participants' emotional well-being (Yang and Ma, 2020). Another explanation is that broadening knowledge about the pandemic outbreak enhances the sense of control and builds participants' emotional well-being (Yang and Ma, 2020).

It is also possible that looking up and talking about COVID-19 as well as engaging in creative activities serve as emotion regulation strategies. It is well-established that young adults manage their moods *inter alia* by seeking others, keeping oneself busy, and being involved in enjoyable activities (Diefendorff et al., 2008). Reading new information about the pandemic, exchanging them with friends and family, and creating could serve as strategies to boost moods. Moreover, to combat boredom, people apply such emotion regulation strategies as “keep myself busy working on other things” and “do something enjoyable to improve my mood” (Diefendorff et al., 2008), which may explain how paradoxically creative activity occurs somehow in pandemic times. Our findings may also be explained by the finding that creativity often serves as a strategy to relieve boredom (Gasper and Middlewood, 2014). Perhaps, breaking the boredom by creative activity is the source of satisfaction.

We do not know whether, while looking for information about the pandemic, the participants did not search for other content that might improve their mood. It is possible that their overall activity in the media, not only to searching for information about the virus but also more entertaining content, could lead to more positive emotions (e.g., Greenwood and Long, 2009). It is also difficult to rule out the possibility that participants' positive emotions resulted from the widespread belief that young people are at a relatively low risk of health complications from COVID-19. This kind of information, as well as social comparison with those who have more demanding situations (e.g., have to take care of balancing work duties with children's home education and facing health problems), could indeed be quite comforting (see Smith, 2000).

## Limitations and Future Research

Although the present studies are micro-longitudinal and use data collected before and during pandemic assessment, still, the findings we present should be interpreted in light of four limitations.

The first is a lack of knowledge about variables, which could help better understand the difference in reaction to information about COVID-19. In future research, it is worth to consider both individual differences (such as personality) and life situation (living alone or sharing household with others, and socioeconomic status) as well as to collect more data about overall media consumption and take a closer look at the content of materials about the pandemic that participants access.

Second, this research did not include reasons for engaging in everyday creativity (see Benedek et al., 2020). In a pandemic situation, personal creativity may rely on further intrinsic motives, such as coping strategies. Future research should thus explore differences in the structure and strength motives in creative activities in situations of distress, such as the COVID-19 pandemic. One possibility would be to use an end-of-day diary design that asks about experiences and activities during the day, using both rating scales (just as in our study) and qualitative responses on the nuances of the activity, motives, and context.

Third, we acknowledge that while our measure of searching for information about the coronavirus captured the intensity, it missed the content, context, and reasons for searching for such information. Therefore, it is possible that, in fact, some people who declared intensive search and contact with COVID-19-related news read something about the possible discovery of new vaccines—unlikely on March–April 2020 yet discussed in media—while some were focused on increasing rates of deaths. That might explain the positive effects of COVID-19 information on both positive and negative emotions. We acknowledge that this lack of differentiation makes the interpretations of our findings challenging.

Fourth and finally, we acknowledge issues associated with our measure of daily creative activity. It was self-report and addressed activity that ranged from everyday, to artistic, to scientific domains. In this study, we were not interested in possible domain-specific effects, so we restrained from a more detailed analysis on the level of particular domains. However, the effects may be more nuanced and better visible in some domains than in others. We invite fellow researchers to use the dataset provided to test additional hypotheses omitted in this study.

## Conclusion

To conclude, although the COVID-19 pandemic is devastating for health and economy and very likely for creative thinking, its impact on creative activity, especially when people are on lockdown, seems to be somehow beneficial. Saying that lockdown is creative would be an overkill. Still, however, as this study demonstrated, there are some positive aspects of thinking and talking about the recent pandemic for young people's creative activities.

## Data Availability Statement

The datasets presented in this study can be found in online repository: https://osf.io/qpzhy/.

## Ethics Statement

The studies involving human participants were reviewed and approved by Institute of Psychology, University of Wrocław, Institutional Review Board. The participants provided their written informed consent to participate in this study.

## Author Contributions

MK: conceptualization, data collection, data analysis, and writing (first draft). AZ: writing and editing. DJ, IO, and ES: data collection, and editing the final draft. IL: data collection, writing and editing. All authors contributed to the article and approved the submitted version.

## Conflict of Interest

The authors declare that the research was conducted in the absence of any commercial or financial relationships that could be construed as a potential conflict of interest.

## References

[B1] Aaker J. Drolet A. Griffin D. (2008). Recalling mixed emotions. J. Consum. Res. 35, 268–278. 10.1086/588570

[B2] Amabile T. M. Barsade S. G. Mueller J. S. Staw B. M. (2005). Affect and creativity at work. Adm. Sci. Q. 50, 367–403. 10.2189/asqu.2005.50.3.367

[B3] Anderson R. Karwowski M. (2020). Motivation and creativity, in Encyclopedia of Creativity, 3rd Edn eds Runco M. Pritzker S. (San Diego, CA: Elsevier), 185–189).

[B4] Baas M. De Dreu C. K. W. Nijstad B. A. (2008). A meta-analysis of 25 years of mood-creativity research: hedonic tone, activation, or regulatory focus? Psychol. Bull. 134, 779–806. 10.1037/a0012815 18954157

[B5] Baas M. De Dreu C. K. W. Nijstad B. A. (2011). Creative production by angry people peaks early on, decreases over time, and is relatively unstructured. J. Exp. Soc. Psychol. 47, 1107–1115. 10.1016/j.jesp.2011.05.009

[B6] Barbot B. Tinio P. P. L. (2015). Where is the “g” in creativity? A specialization–differentiation hypothesis. Front. Hum. Neurosci. 8:1041. 10.3389/fnhum.2014.01041 25628551 PMC4292771

[B7] Barrett L. F. Russell J. A. (1999). The structure of current affect. Curr. Direct. Psychol. Sci. 8, 10–14. 10.1111/1467-8721.00003

[B8] Beghetto R. A. (2020). How times of crisis serve as a catalyst for creative action: an agentic perspective. Front. Psychol. 10.3389/fpsyg.2020.600685 33488463 PMC7817941

[B9] Benedek M. Bruckdorfer R. Jauk E. (2020). Motives for creativity: exploring the what and why of everyday creativity. J. Creat. Behav. 54, 610–625. 10.1002/jocb.396

[B10] Benedek M. Jauk E. Kerschenbauer K. Anderwald R. Grond L. (2017). Creating art: an experience sampling study in the domain of moving image art. Psychol. Aesthet. Creat. Arts 11, 325–334. 10.1037/aca0000102

[B11] Bledow R. Rosing K. Frese M. (2013). A dynamic perspective on affect and creativity. Acad. Manag. J. 56, 432–450. 10.5465/amj.2010.0894

[B12] Carson S. H. Peterson J. B. Higgins D. M. (2005). Reliability, validity, and factor structure of the creative achievement questionnaire. Creat. Res. J. 17, 37–50. 10.1207/s15326934crj1701_4

[B13] Comunian R. England L. (2020). Creative and cultural work without filters: Covid-19 and exposed precarity in the creative economy. Cultural Trends 29, 112–128. 10.1080/09548963.2020.1770577

[B14] Conner T. S. DeYoung C. G. Silvia P. J. (2018). Everyday creative activity as a path to flourishing. J. Posit. Psychol. 13, 181–189. 10.1080/17439760.2016.1257049

[B15] Conner T. S. Silvia P. J. (2015). Creative days: a daily diary study of emotion, personality, and everyday creativity. Psychol. Aesthet., Creat. Arts 9, 463–470. 10.1037/aca0000022

[B16] Creative Industries Federation (2020). Press Release: The Projected Economic Impact of Covid-19 on the UK Creative Industries Report. Available online at: https://www.creativeindustriesfederation.com/news/press-release-cultural-catastrophe-over-400000-creative-jobs-could-be-lost-projected-economic (accessed August 23, 2020).

[B17] Davis M. A. (2009). Understanding the relationship between mood and creativity: a meta-analysis. Org. Behav. Hum. Decis. Process. 108, 25–38. 10.1016/j.obhdp.2008.04.001

[B18] Diefendorff J. M. Richard E. M. Yang J. (2008). Linking emotion regulation strategies to affective events and negative emotions at work. J. Vocat. Behav. 73, 498–508. 10.1016/j.jvb.2008.09.006

[B19] Dollinger S. J. (2007). Creativity and conservatism. Pers. Indiv. Differ. 43, 1025–1035. 10.1016/j.paid.2007.02.023

[B20] Duan H. Wang X. Hu W. Kounios J. (2020). Effects of acute stress on divergent and convergent problem-solving. Think. Reason. 26, 68–86. 10.1080/13546783.2019.1572539

[B21] Fischer R. Karl J. Bortolini T. Robinson K. André Luiz Alves Rabelo A. L. A. Trâm N. T. B.. (2020). Rapid review and meta-meta-analysis of self-guided interventions to address anxiety, depression and stress during COVID-19 social distancing. Front. Psychol. 11:563876. 10.3389/fpsyg.2020.563876 33192837 PMC7655981

[B22] Fong C. T. (2006). The effects of emotional ambivalence on creativity. Acad. Manag. J. 49, 1016–1030. 10.5465/amj.2006.22798182

[B23] Friedman R. S. Förster J. Denzler M. (2007). Interactive effects of mood and task framing on creative generation. Creat. Res. J. 19, 141–162. 10.1080/10400410701397206

[B24] Gasper K. Middlewood B. L. (2014). Approaching novel thoughts: understanding why elation and boredom promote associative thought more than distress and relaxation. J. Exp. Soc. Psychol. 52, 50–57. 10.1016/j.jesp.2013.12.007

[B25] George J. M. Zhou J. (2002). Understanding when bad moods foster creativity and good ones don't: the role of context and clarity of feelings. J. Appl. Psychol. 87, 687–697. 10.1037/0021-9010.87.4.687 12184573

[B26] Greenwood D. N. Long C. R. (2009). Mood specific media use and emotion regulation: Patterns and individual differences. Pers. Indiv. Differ. 46, 616–621. 10.1016/j.paid.2009.01.002

[B27] Groyecka A. (2018). Will becoming more creative make us more tolerant? Creat. Theor. Res. Appl. 5, 170–176. 10.1515/ctra-2018-0015

[B28] Han W. Feng X. Zhang M. Peng K. Zhang D. (2019). Mood states and everyday creativity: employing an experience sampling method and a day reconstruction method. Front. Psychol. 10:1698. 10.3389/fpsyg.2019.01698 31379699 PMC6658875

[B29] Hobfoll S. E. (2001). The influence of culture, community, and the nested-self in the stress process: advancing conservation of resources theory. Appl. Psychol. 50, 337–421. 10.1111/1464-0597.00062

[B30] Hobfoll S. E. Halbesleben J. Neveu J.-P. Westman M. (2018). Conservation of resources in the organizational context: the reality of resources and their consequences. Annu. Rev. Org. Psychol. Org. Behav. 5, 103–128. 10.1146/annurev-orgpsych-032117-104640

[B31] Ivcevic Z. (2007). Artistic and everyday creativity: An act-frequency approach. J. Creat. Behav. 41, 271–290. 10.1002/j.2162-6057.2007.tb01074.x

[B32] Ivcevic Z. Mayer J. D. (2009). Mapping dimensions of creativity in the life-space. Creat. Res. J. 21, 152–165. 10.1080/10400410902855259

[B33] Jankowska D. M. Czerwonka M. Lebuda I. Karwowski M. (2018). Exploring the creative process: integrating psychometric and eye-tracking approaches. Front. Psychol. 9:1931. 10.3389/fpsyg.2018.01931 30356907 PMC6190897

[B34] Kapoor H. Kaufman J. C. (2020). Meaning-making through creativity during COVID-19. Front. Psychol. 11:595990 10.3389/fpsyg.2020.595990 33391115 PMC7775492

[B35] Karwowski M. (2016). The dynamics of creative self-concept: changes and reciprocal relations between creative self-efficacy and creative personal identity. Creat. Res. J. 28, 99–104. 10.1080/10400419.2016.1125254

[B36] Karwowski M. Brzeski A. (2017). Selfies and the (creative) self: a diary study. Front. Psychol. 8:172. 10.3389/fpsyg.2017.00172 28228743 PMC5296334

[B37] Karwowski M. Groyecka-Bernard A. Kowal M. Sorokowski P. (2020). Does thinking about coronavirus impact insight and analytical reasoning? Think. Skills Creat. 38:100715. 10.1016/j.tsc.2020.100715 32843905 PMC7431327

[B38] Karwowski M. Lebuda I. Szumski G. Firkowska-Mankiewicz A. (2017). From moment-to-moment to day-to-day: experience sampling and diary investigations in adults' everyday creativity. Psychol. Aesthet. Creat. Arts 11, 309–324. 10.1037/aca0000127

[B39] Karwowski M. Wiśniewska E. (in press). Creativity in adulthood, in The Cambridge Handbook of Lifespan Development of Creativity, eds Hoffmann J. D. Russ S. W. Kaufman J. C. (Cambridge University Press).

[B40] Kaufman J. C. Beghetto R. (2009). Beyond big and little: the four c model of creativity. Rev. Gen. Psychol. 13, 1–12. 10.1037/a001368833391115

[B41] Kaufmann G. Vosburg S. K. (2002). The effects of mood on early and late idea production. Creat. Res. J. 14, 317–330. 10.1207/S15326934CRJ1434_3

[B42] Kowal M. Coll-Martín T. Ikizer G. Rasmussen J. Eichel K. Studzinska A.. (2020). Who is the most stressed during the COVID-19 pandemic? Data from 26 countries and areas. Appl. Psychol. Health Well Being. 12, 946–966. 10.1111/aphw.12234 32996217 PMC7537225

[B43] Mercier M. Vinchon F. Pichot N. Bonetto E. Bonnardel N. Girandola F. (2021). COVID-19: a boon or a bane for creativity? Front. Psychol. 11:601150 10.3389/fpsyg.2020.601150 33536973 PMC7848087

[B44] Middlewood B. L. Gallegos J. Gasper K. (2016). Embracing the unusual: feeling tired and happy is associated with greater acceptance of atypical ideas. Creat. Res. J. 28, 310–317. 10.1080/10400419.2016.1195639

[B45] Moeller J. Ivcevic Z. Brackett M. A. White A. E. (2018). Mixed emotions: network analyses of intra-individual co-occurrences within and across situations. Emotion 18, 1106–1121. 10.1037/emo0000419 29389203

[B46] Muthén L. K. Muthén B. O. (1998–2017). Mplus User's Guide, 8th Edn Los Angeles, CA: Muthén & Muthén.

[B47] Nezlek J. B. (2008). An introduction to multilevel modeling for social and personality psychology. Soc. Pers. Psychol. Compass 2, 842–860. 10.1111/j.1751-9004.2007.00059.x

[B48] Nezlek J. B. (2011). Multilevel Modeling for Social and Personality Psychology. Newbury Park, CA: SAGE Publications Ltd.

[B49] Orkibi H. (2020). Creative adaptability: conceptual framework, measurement, and outcomes in times of crisis. Front. Psychol. 11:588172. 10.3389/fpsyg.2020.588172 33510671 PMC7835130

[B50] Pérez-Fuentes M. C. Molero Jurado M. M. Martos Martínez Á. Gázquez Linares J. J. (2020). Threat of COVID-19 and emotional state during quarantine: positive and negative affect as mediators in a cross-sectional study of the Spanish population. PLOS ONE 15:e0235305. 10.1371/journal.pone.0235305 32584897 PMC7316299

[B51] Rees L. Rothman N. B. Lehavy R. Sanchez-Burks J. (2013). The ambivalent mind can be a wise mind: Emotional ambivalence increases judgment accuracy. J. Exp. Soc. Psychol. 49, 360–367. 10.1016/j.jesp.2012.12.017

[B52] Richards R. (2010). Everyday creativity: process and way of life – four key issues, in The Cambridge Handbook of Creativity, eds Kaufman J. C. Sternberg R. J. (New York, NY: Cambridge University Press), 189–215.

[B53] Shanahan L. Steinhoff A. Bechtiger L. Murray A. L. Nivette A. Hepp U.. (2020). Emotional distress in young adults during the COVID-19 pandemic: evidence of risk and resilience from a longitudinal cohort study. Psychol. Med. 1–10. 10.1017/S003329172000241X 32571438 PMC7338432

[B54] Silvia P. J. Beaty R. E. Nusbaum E. C. Eddington K. M. Levin-Aspenson H. Kwapil T. R. (2014). Everyday creativity in daily life: an experience-sampling study of “little c” creativity. Psychol. Aesthet. Creat. Arts 8, 183–188. 10.1037/a0035722

[B55] Smith R. H. (2000). Assimilative and contrastive emotional reactions to upward and downward social comparisons, in Handbook of social comparison, eds Suls J. Wheller L. (Plenum), 173–200.

[B56] Sorokowski P. Groyecka A. Kowal M. Sorokowska A. Białek M. Lebuda I. (2020). Can Information about pandemics increase negative attitudes toward foreign groups? A case of COVID-19 outbreak. Sustainability 12:4912 10.3390/su12124912

[B57] Van Kleef G. A. Anastasopoulou C. Nijstad B. A. (2010). Can expressions of anger enhance creativity? A test of the emotions as social information (EASI) model. J. Exp. Soc. Psychol. 46, 1042–1048. 10.1016/j.jesp.2010.05.015

[B58] Webster D. M. Kruglanski A. W. (1994). Individual differences in need for cognitive closure. J. Pers. Soc. Psychol. 67, 1049–1062. 10.1037/0022-3514.67.6.1049 7815301

[B59] Yang H. Ma J. (2020). How an epidemic outbreak impacts happiness: factors that worsen (vs. protect) emotional well-being during the coronavirus pandemic. Psychiatry Res. 289:113045. 10.1016/j.psychres.2020.113045 32388418 PMC7190485

[B60] Zielińska A. (2020). Mapping adolescents' everyday creativity. Creat. Theor. Res. Appl. 7, 208–229. 10.2478/ctra-2020-0012

