# Supplementary material for: Creative Lockdown? A Daily Diary Study of Creative Activity During Pandemics
Source: Front Psychol. 2021 Feb 9;12:600076. doi: 10.3389/fpsyg.2021.600076 (PMC7900138; doi:10.3389/fpsyg.2021.600076)
Supplement: Supplementary file 1 [file Table_1.docx]

**Supplementary Online Material for**

**Creative Lockdown?**

**A Daily Diary Study of Creative Activity During Pandemics**

SOM T1. Descriptive Statistics and Factor Structure for Creative Activity and Emotions

|  | *M* | *SD* | F1 | F2 |
| --- | --- | --- | --- | --- |
| Creativity |  |  |  |  |
| Writing, e.g. poetry, short stories, novels, theatrical plays | 1.40 | 1.00 |  |  |
| Writing press articles (including e.g. columns) | 1.09 | 0.45 |  |  |
| Writing scholarly papers | 1.23 | 0.74 |  |  |
| Preparing for public speeches / giving public speeches | 1.29 | 0.84 |  |  |
| Creating online blog(s) entries | 1.19 | 0.67 |  |  |
| Composing musical pieces / playing music | 1.36 | 0.93 |  |  |
| Designing clothing items | 1.15 | 0.64 |  |  |
| Designing buildings / interiors | 1.22 | 0.75 |  |  |
| Cooking based on one’s own recipes | 2.18 | 1.49 |  |  |
| Painting / drawing / sculpting | 1.51 | 1.10 |  |  |
| Creating choreographies / Dancing | 1.29 | 0.85 |  |  |
| Taking photos / making videos, e.g. with a phone | 1.97 | 1.34 |  |  |
| Solving technical / scientific problems | 1.46 | 0.99 |  |  |
| Programing / creating computer programs | 1.05 | 0.35 |  |  |
| Creating websites | 1.03 | 0.27 |  |  |
| Emotions |  |  |  |  |
| Interested | 3.13 | 1.12 | -.23 | **.71** |
| Bored | 2.36 | 1.20 | **.49** | -.25 |
| Confused | 1.85 | 1.07 | **.60** | .12 |
| Surprised | 2.31 | 1.21 | .16 | **.61** |
| Interested | 3.26 | 1.10 | -.23 | **.70** |
| Afraid | 1.67 | 1.04 | **.64** | .10 |
| Frustrated | 2.19 | 1.27 | **.76** | -.14 |
| Thought-provoked | 3.19 | 1.17 | -.15 | .**58** |
| Dull | 1.82 | 1.12 | .**58** | -.20 |
| Amazed | 1.83 | 1.04 | .26 | **.56** |
| Concerned | 2.12 | 1.21 | **.71** | .02 |
| Happy | 3.33 | 1.14 | -.47 | **.63** |
| Besotted | 1.70 | 1.03 | **.59** | -.09 |
| Annoyed | 2.23 | 1.24 | **.75** | -.10 |
| Sleepy | 2.36 | 1.29 | **.44** | -.13 |
| Excited | 2.38 | 1.24 | -.14 | **.71** |
| Amazed | 1.92 | 1.06 | .26 | **.58** |
| Dissatisfied | 2.09 | 1.18 | .**78** | -.18 |
| Nervous | 2.18 | 1.23 | .**77** | -.08 |
| Joyful | 3.21 | 1.16 | -.48 | **.64** |
| Puzzled | 1.74 | 1.01 | **.62** | .15 |

*Note.* *N* = 6,821 (day level). All items were measure on 1-5 Likert Scale.
